# Research on Monocrystalline Silicon Micro-Nano Structures Irradiated by Femtosecond Laser

**DOI:** 10.3390/ma15144897

**Published:** 2022-07-14

**Authors:** Yanan Liu, Ye Ding, Jichang Xie, Mingjun Chen, Lijun Yang, Xun Lv, Julong Yuan

**Affiliations:** 1 School of Mechatronics Engineering, Harbin Institute of Technology, Harbin 150001, China; tjgdlyn@163.com (Y.L.); chenmj@hit.edu.cn (M.C.); 2 Key Laboratory of Micro-Systems and Micro-Structures Manufacturing, Ministry of Education, Harbin Institute of Technology, Harbin 150001, China; 3 Laboratoire Roberval, UTC, Sorbonne Universités, Université de Technologie de Compiègne, Centre de Recherche Royallieu, CS60319, CEDEX, 60203 Compiègne, France; xjctjpu@163.com; 4 Xinchang Research Institute of Zhejiang University of Technology, Xinchang 312500, China; lvxun@zjut.edu.cn (X.L.); jlyuan@zjut.edu.cn (J.Y.); 5 College of Mechanical Engineering, Zhejiang University of Technology, Hangzhou 310014, China

**Keywords:** femtosecond laser, monocrystalline silicon, laser-induced periodic surface structure, micro-nano structure

## Abstract

Femtosecond (fs) laser processing has received great attention for preparing novel micro-nano structures and functional materials. However, the induction mechanism of the micro-nano structures induced by fs lasers still needs to be explored. In this work, the laser-induced periodic surface structure (LIPSS) of monocrystalline silicon (Si) under fs laser irradiation is investigated. Three different layers named amorphous silicon (a-Si) layer, transition layer, and unaffected Si layer are observed after laser irradiation. The a-Si layer on the surface is generated by the resolidification of melting materials. The unaffected Si layer is not affected by laser irradiation and maintains the initial atomic structure. The transition layer consisting of a-Si and unaffected Si layers was observed under the irradiated subsurface. The phase transition mechanism of Si irradiated by fs laser is “amorphous transition”, with the absence of other crystal structures. A numerical model is established to describe the fs laser-Si interaction to characterize the electronic (lattice) dynamics of the LIPSS formation. The obtained results contribute to the understanding of fs laser processing of Si at the atomic scale as well as broaden the application prospects of fs laser for treating other semiconductor materials.

## 1. Introduction

Monocrystalline silicon (Si) is the most widely used first-generation semiconductor and has been applied in electronics, biology, energy, and communication, among other areas [1]. The fabrication of micro-nano structures on Si surfaces is a crucial factor in these applications. To date, a variety of micro-and nano-fabrication techniques have been established [2,3,4], including electron beam lithography, additive manufacturing, plasma etching, and focused ion beam processing. However, smooth surfaces, complex operations, and expensive equipment are commonly required in these techniques, making the rapid production of Si nanostructured surfaces challenging. Femtosecond (fs) laser, a promising high-resolution material processing technology, has been proven to be an ideal tool for regulating the surface energy deposition and inducing phase transitions of materials [5]. The foremost advantages of fs laser are the rapid energy transfer and limited heat-affected areas, which suggest great potential in the field of micro-and nano-scale manufacturing. Various surface structures induced by fs-laser on numerous materials have been reported, including semiconductors, metals, dielectrics, ceramics, and polymers [6,7,8,9,10]. It is obvious that the fs laser plays an essential role in photonics, biomedicine, and nanomanufacturing fields [11,12].

In recent decades, laser-induced periodic surface structuring (LIPSS) has been reported to fabricate different materials, which has attracted extensive attention in the field of ultrafast laser micro-nano processing [13,14,15,16,17,18]. Concerning the potential physical origin of LIPSS formation, various mechanisms have been proposed to explain its generation, such as interference between the laser and induced scattering wave, or with the surface plasmon polariton (SPP), self-organization mechanisms, Marangoni convection flow, Marangoni bursting, and liquid vortexes and flows, etc. [19,20,21,22,23,24]. Although the exact mechanism of the origin of the LIPSS remains under debate, there is no doubt that the fs laser irradiation process eventually leads to modification of the material surfaces. Fs laser irradiation can enable the materials to enter an extreme nonequilibrium state and trigger a series of structural changes, resulting in complex multi-scale surface morphology, unusual metastable phase, and microstructures that are difficult to produce by other methods [25]. Recent studies have revealed that the extremely high-pressure and high-temperature conditions generated by fs laser-induced confined micro-explosions realize a new metastable tetragonal phase of Si, providing a favorable pathway for opening up a series of new metastable phases, and expanding the further exploration of new crystal structure materials [26]. Furthermore, fs laser irradiation can cause rapid heating and melting of the material surface or internal local area and produce a high cooling rate of more than 10^12^ K/s, leading to instantaneous melting and rapid quenching of materials [27]. The extraordinarily nonequilibrium ultrafast process depends on the interaction between photons and electrons, which directly determines the shape and performance of the final structure, thus determining the function of the device. Therefore, research on the interaction of fs lasers with Si is necessary to broaden the understanding of the behavior of materials under extreme conditions and identify the generation of new phase transitions and microstructures [28].

The interaction between fs laser and semiconductors is a complex process involving energy transport and electron (lattice) dynamics across time and space scales [29]. During fs laser irradiation on Si materials, a thin layer on its surface undergoes ultrafast melting and resolidification processes, resulting in atomic positions and structural properties changes [30]. In addition, the extremely nonequilibrium characteristic of fs laser processing leads to amorphization, high-pressure phases, self-organizing structure, nanocrystalline phases, and pressure-driven phase transitions of Si [31]. Nevertheless, up to now, the micro-nano structures of Si induced by fs laser are yet to be in-depth investigated at the atomic scale.

To this end, the fs laser–Si interactions, LIPSS formation, and the phase transition mechanisms involved during fs laser processing were investigated through both experimental and numerical approaches. TEM (transmission electron microscope) characterization was employed to analyze the phase transition and interface structure of the LIPSS on the atomic scale. The characteristics and formation mechanisms of amorphous silicon (a-Si) preferentially formed in the surface layer and the peak center were determined. Simultaneously, a theoretical model was developed for simulating the ultrafast thermal and optical dynamics of Si with fs laser irradiation. Based on these results, the phase transition and ultrafast mechanisms of Si during LIPSS formation were proposed. This work focuses on the TEM characterization of Si micro-nano structures irradiated by fs laser and elucidates the electronic (lattice) dynamics involved in the ablation process based on numerical simulations. It aims to provide a new perspective of fs laser processing for the preparation of functional micro-nano structure surfaces and materials.

## 2. Theoretical Background

In this section, a theoretical model was established to simulate the electron and lattice dynamics of Si during the fs laser irradiation. The parameters of Si used in the model are listed in Table 1 [32]. Considering carrier generation, carrier diffusion, and Auger recombination, the change in the carrier density (*N_e_*) as a function of time in the conduction band (CB) is described by a nonlinear partial differential equation (Equation (1)). The electron (*T_e_*) and lattice (*T_l_*) temperature are defined by the two-temperature model (TTM) based on Equations (2) and (3) [33,34,35]:

(1) $$\frac{\partial N_{e}}{\partial t}=\nabla \left(k_{B}T_{e}\mu_{e}\nabla N_{e}\right)+J_{e}\frac{n_{0}-N_{e}}{n_{0}}-P_{e}$$

(2) $$C_{e}\frac{\partial T_{e}}{\partial t}=K_{e}\nabla^{2}T_{e}-ϒ\left(T_{e}-T_{l}\right)+Q$$

(3) $$C_{l}\frac{\partial T_{l}}{\partial t}=K_{l}\nabla^{2}T_{l}+ϒ\left(T_{e}-T_{l}\right)$$

where *k_B_* is the Boltzmann constant, *μ_e_* is the carrier mobility, *J_e_* is the gain of free carriers per unit time and volume, *n*_0_ denotes the atomic density of non-excited Si, and *P_e_* represents the conduction electron loss caused by Auger recombination. In the TTM model, *t* is time, ϒ is the coupling coefficient between the electron and the lattice, *C_e_* (*C_l_*) and *K_e_* (*K_l_*) represent the electron (lattice) heat capacity and electron (lattice) thermal conductivity of Si, respectively. The heat source term (*Q*) contains one-photon and two-photon absorption, the electron energy loss caused by impact ionization, the free-carrier heating, and the energy released by Auger recombination [36], which can be expressed as follows:

(4) $$Q=\left[\left(\hbar \omega -E_{0}\right)\frac{\psi_{1}I}{\hbar \omega }+\left(2\hbar \omega -E_{0}\right)\frac{\psi_{2}I^{2}}{2\hbar \omega }-\delta E_{0}N_{e}\right]\frac{n_{0}-N_{e}}{n_{0}}+\alpha I+E_{0}P_{e}-\frac{3}{2}k_{B}T_{e}\frac{\partial N_{e}}{\partial t}$$

where ω is the laser angular frequency, ħ is the reduced Planck constant, $\psi_{1}$ and $\psi_{2}$ are the one- and two-photon absorption coefficients, *E*_0_ is the bandgap, δ is the impact ionization coefficient, and *α* is the free electron absorption coefficient. The laser intensity on the Si surface is $I=\left(1-R\right)I_{E}$ , *R* denotes the surface reflectivity. The energy attenuation of the laser beam propagating inside the sample is determined by the multiphoton loss and inverse Bremsstrahlung absorption [32], which is expressed as:

(5) $$\frac{dI}{dz}=-\psi_{1}I-\psi_{2}I^{2}-\alpha I$$

where *z* is the depth into the sample, and absorption coefficient *α* is calculated via:

(6) α=2ωcIm1−ωp2/ω2(1+ivω)

where *c* is the light speed, $\omega_{p}$ is the plasma frequency, and v denotes the carrier collision frequency.

To explain the phenomenon of modulation and absorption of fs laser pulse caused by the interference between the electromagnetic wave and the excited laser field on the Si surface, the periodic disturbance is added to the lateral intensity distribution of the laser beam. The incident laser intensity *I_E_* is given by [37]:

(7) $$I_{E}=\frac{2F_{I}}{t_{p}}\sqrt{\frac{\ln2}{\pi }}\left[1+\zeta \cos(\frac{2\pi x}{\Omega_{x}})\right]\exp\left[\frac{-2\left(x^{2}+y^{2}\right)}{R_{0}^{2}}\right]\exp\left[-4\ln2{\frac{\left(t-t_{p}\right)}{t_{p}^{2}}}^{2}\right]$$

where *F_I_* = 2*E_p_*/(*πR*_0_)^2^ denotes the pulse fluence, *E_p_* stands for the pulse energy, *R*_0_ is the laser spot radius, *t_p_* is the laser pulse duration, and $\Omega_{x}$ represents the spatial period, which is regarded as approximately equal to the wavelength of the surface electromagnetic wave Λ*_E_* and is expressed as follows [38]:

(8) Ωx∼ΛE=λ/(Re(εε+1)±sinθ)

where *λ* is the laser wavelength, *θ* is the laser incident angle, and *ε* is the dielectric permittivity.

The free electrons in the Si valence band (VB) absorb photons and transit to the CB induced by the fs laser irradiation. The dielectric permittivity *ε* can be expressed by using the Drude model [39]:

(9) $$\varepsilon =1+\left(\varepsilon_{Si}-1\right)\left(1-\frac{N_{e}}{N_{0}}\right)-\frac{\omega_{p}^{2}\left(N_{e}\right)}{\omega^{2}\left(1+i\frac{\nu }{\omega }\right)}$$

where $\varepsilon_{Si}$ = 13.64 + 0.048*i* is the dielectric constant of non-excited Si. *N*_0_ stands for the total valance band density.

The reflection of Si on the fs laser heat source directly affects the electron–lattice nonequilibrium heat transfer and laser energy deposition process, which can be written as Equations (10)–(12) [36]:

(10) $$n=n_{1}+in_{2}$$

(11) $$n_{1}=\sqrt{\frac{\varepsilon_{1}+\sqrt{\varepsilon_{1}^{2}+\varepsilon_{2}^{2}}}{2}}$$

(12) $$n_{2}=\sqrt{\frac{-\varepsilon_{1}+\sqrt{\varepsilon_{1}^{2}+\varepsilon_{2}^{2}}}{2}}$$

where *n*_1_ is the normal refractive index, and *n*_2_ denotes the extinction coefficient. *ε*_1_ and *ε*_2_ represent the real and imaginary parts of the dielectric coefficient.

Based on the above equations, the reflection *R* of the Si surface can be calculated:

(13) $$R=\frac{{\left(n_{1}-1\right)}^{2}+n_{2}^{2}}{{\left(n_{1}+1\right)}^{2}+n_{2}^{2}}$$

The dimension of the TTM simulation domain is 20 × 20 × 1 mm^3^ with a minimum grid size of 100 nm. The step size was set as 5 fs. The initial values of the system were set as *T_e_* = *T*_l_ = 300 K, $\partial T_{e}/\partial_{t}=0$ , $\partial T_{l}/\partial_{t}=0$ , and the initial electron density (*N_I_*) value of Si was 1 × 10^24^ m^−3^.

## 3. Experimental Processes and Sample Tests

### 3.1. Materials and Laser Irradiation

**Figure 1:**
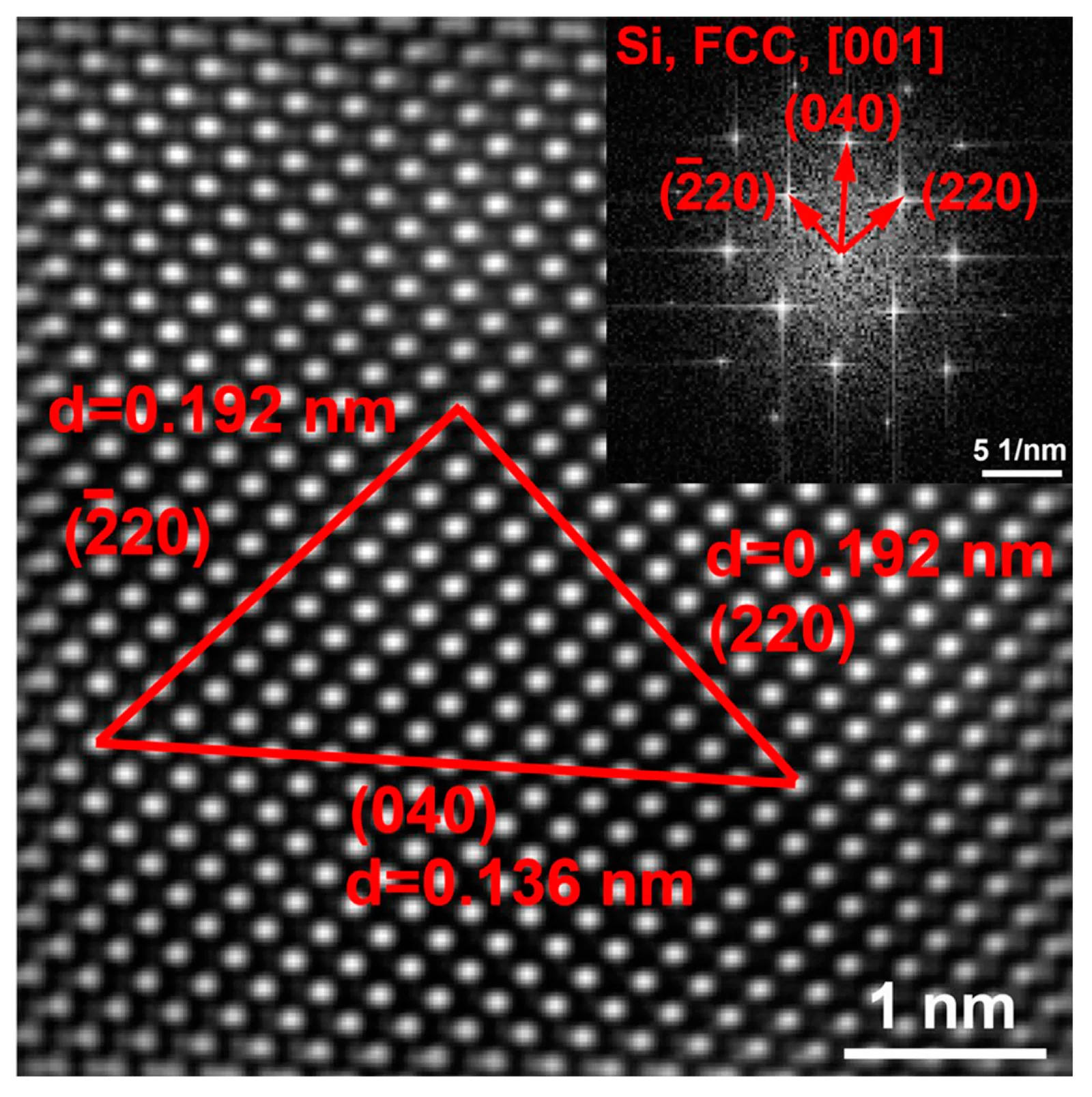
HRTEM image of [1] ZA of the pristine Si. The inset is the corresponding FFT image.

The standard one-side-polished (100) crystallographic Si wafer with a size of 10 × 10 × 0.6 mm^3^ was chosen for single-pulse fs laser irradiation. The high-resolution transmission electron microscope (HRTEM, Talos F200X, FEI Corp., Hillsborough, OR, USA) and corresponding detailed fast Fourier transform (FFT) images of the sample are shown in Figure 1. It is proved that [1] is the dominant exposed zone axis (ZA) and consists of (040), (220), and $\left(\bar{2}20\right)$ lattice planes with the distance of 0.136 nm, 0.192 nm, and 0.192 nm, respectively. A linearly polarized fs laser system (Pharos, Light Conversion Ltd., Vilnius, Lithuania), working at the 1030 nm wavelength with a pulse duration of 300 fs and a repetition rate of 1 MHz, was used to irradiate the sample. A pulse picker divider is integrated into the used laser source. Its *n*_1_ value indicates that one of *n*_1_ pulses in the pulse train is selected to be emitted from the laser source. In addition, the laser machining system is equipped with a high-speed optical shutter (10FBS-5-10, Micro photons Technology, Co., Ltd., Shanghai, China), with a maximum working frequency of 2 kHz. By setting the values of shutter open time, shutter working frequency, *n*_1_, and laser repetition rate as 1, 1 kHz, 1000, and 1 MHz, respectively, the effective emitting laser pulse number is calculated as 1 ÷ 1000 ÷ 1000 × 10^6^ = 1. Therefore, the single pulse laser irradiation strategy can be realized at a fixed position. The laser beam was focused on the sample surface through an optical lens with a focal length of 50 mm, and the focal spot diameter was approximately 11 μm. The sample was fixed on a three-dimensional micro-mobile platform with a positioning accuracy of 1 um for processing in an atmospheric environment. After laser processing, the obtained sample was placed in ethanol for ultrasonic cleaning for 15 min.

### 3.2. Characterization

The phase composition of the pristine Si was characterized using X-ray diffraction (XRD, X’PERT, Panalytical, Almelo, The Netherlands) in the range of 10°–100° at a scanning rate of 5°/min. The internal LIPSS structure was characterized via transmission electron microscopy (TEM, Talos F200X, FEI Corp., Hillsborough, OR, USA) operated at 200 kV and equipped with an energy dispersive X-ray spectrometer (EDS). The TEM sample is extracted by a focused ion beam device (FIB, Helios Nanolab 600i, FEI Corp.) from the center position irradiated by the laser.

## 4. Results and Discussion

### 4.1. Ultrafast Electron and Lattice Dynamics

**Figure 2:**
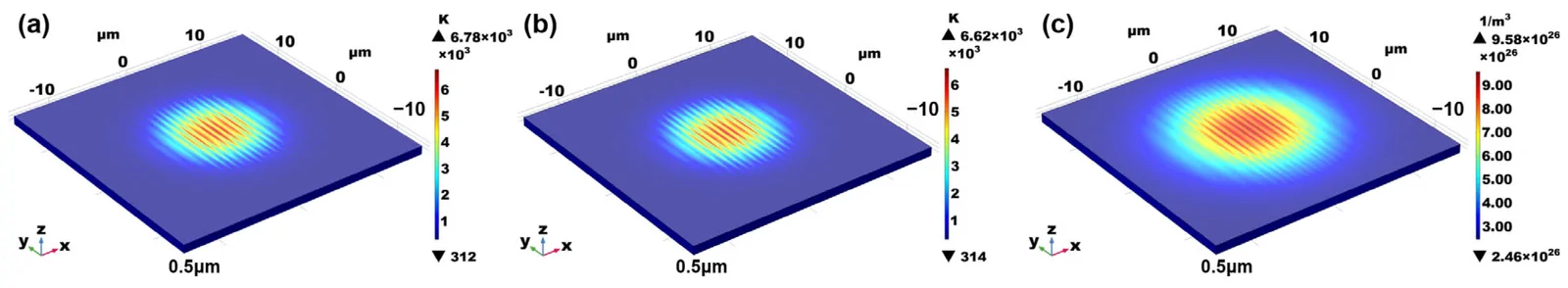
The evolution of the calculated (**a**) *T_e_*, (**b**) *T_l_*, and (**c**) *N_e_* at 12 ps.

**Figure 3:**
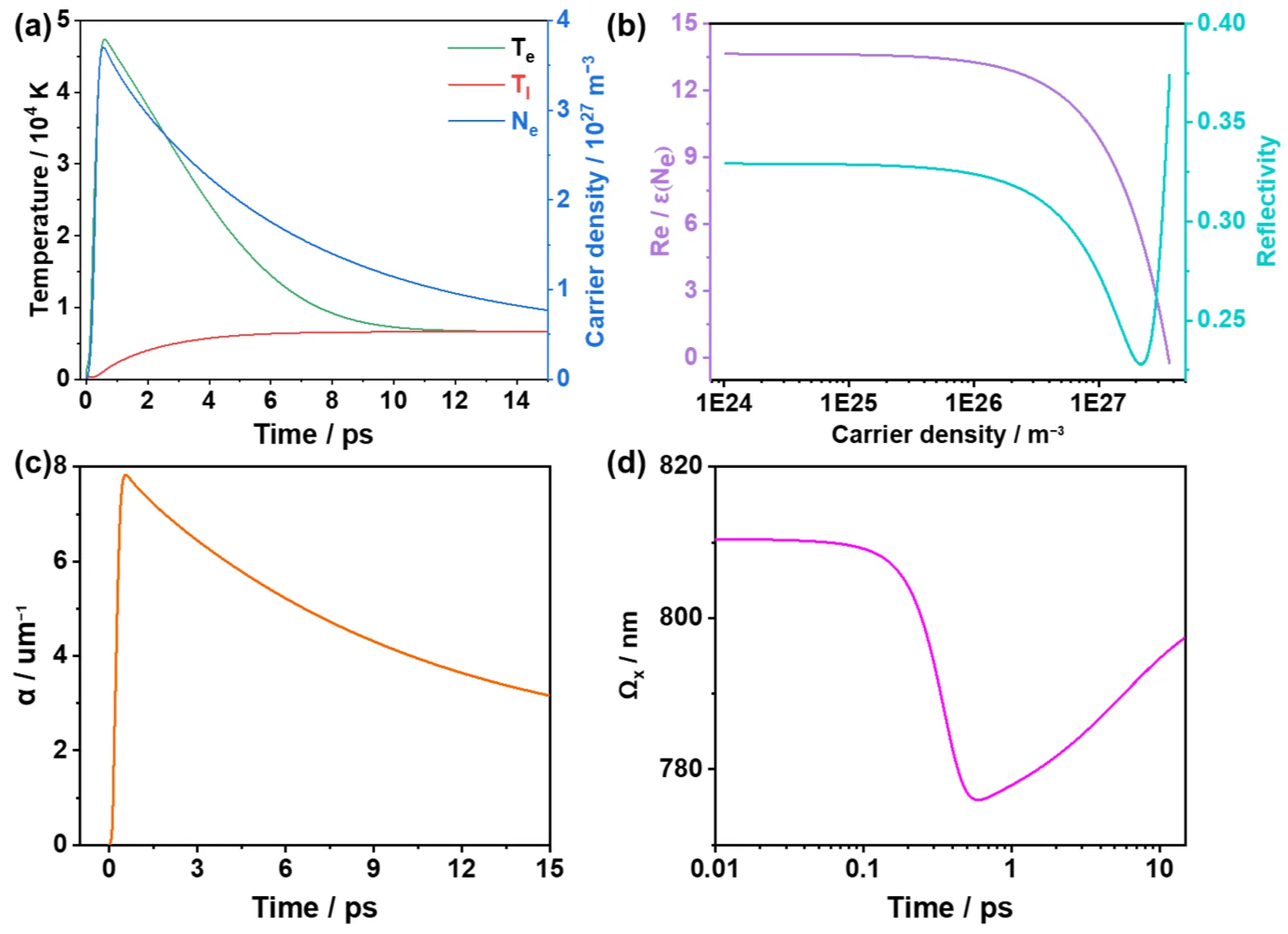
(**a**) Calculated *T_e_*, *T_l_*, and *N_e_* as a function of time; (**b**) the real part of the dielectric constant and reflectivity calculated as a function of carrier density; (**c**) time dependence of the evolution of absorption coefficient; (**d**) wavelength period of SPP as a function of time.

Based on simulating the electron (lattice) dynamics of Si under fs laser irradiation, the evolution of the electron (lattice) temperature and optical properties can be obtained. Figure 2 illustrates the distribution of electrons, lattice temperature, and carrier density of Si at 12 ps under laser irradiation. It can be discovered that the distribution of electrons, lattice temperature, and carrier density of Si exhibit evident periodicity, which is modulated by the periodic interference energy field formed by the incident laser and the SPP. Figure 3a shows the time-dependent evolution of electron (lattice) temperature and carrier density at the x = 0, y = 0 and z = 0 positions. Firstly, the carriers absorb the laser pulse energy in an extremely short time, resulting in a rapid temperature rise to a peak value of 4.73 × 10^4^ K at 600 fs. Meanwhile, the carrier density reached a peak of 3.69 × 10^27^ m^−3^. Subsequently, the thermalized free electrons undergo rapid inelastic collisions with the surrounding lattice, which are transferred to the lattice system through the carrier–lattice coupling effect. The electron temperature and carrier density gradually decrease due to the end of laser incidence. Finally, the electron and lattice temperatures reach thermal equilibrium at 12 ps. Notably, the equilibrium temperature is approximately 6700 K, which is higher than the melting point of Si (1687 K), indicating that the laser fluence has exceeded its melting threshold.

Based on the Drude model, a large number of electrons in the VB are excited by fs laser to form free electrons in the CB. The carrier density change in Si will affect its optical properties [40]. Figure 3b displays the evolution of the real part of the dielectric constant and surface reflectivity of Si. The fs laser irradiation leads to the increasing carrier density. During this process, the reflectivity is reduced with the enhanced absorption of laser energy by Si. With the increase of carrier density, the real part of the dielectric constant gradually decreases and eventually becomes negative, i.e., the optical properties of the Si surface will evolve from a semiconductor to a metal-like material. The resonance absorption is generated when plasma frequency approaches the laser frequency, resulting in the lowest reflectivity. Subsequently, the increased reflectivity indicates a decrease in the energy coupling efficiency between the laser and the highly excited surface. Figure 3c demonstrates that the absorption coefficient increases rapidly in the high excitation state and gradually decreases when the laser pulse disappears. This result reflects that the variations of the reflectivity and absorption coefficient of Si indicate the influence of the carrier density on its optical properties. Furthermore, the wavelength range of the SPP could estimate the expected subwavelength structure on fs laser-irradiated Si. Figure 3d exhibits the period of SPP as a function of time. It is seen that the generation period varies significantly with time and the period of the SPP is in the range of 775–810 nm. Therefore, the energy transfer of fs laser irradiation on Si transmits photon energy to VB electrons through photoionization, causing changes in carrier density to affect the optical properties of Si. In particular, numerous excited free electrons form SPP on the Si surface. The energy field generated by the interference of SPP with the incident laser removes Si through an ultrafast heat effect, resulting in the LIPSS formation. Therefore, the excitation of SPP and its interference with the incident laser beam is regarded as the primary formation mechanism of the LIPSS.

### 4.2. Surface Micro-Nano Structure Characteristics

**Figure 4:**
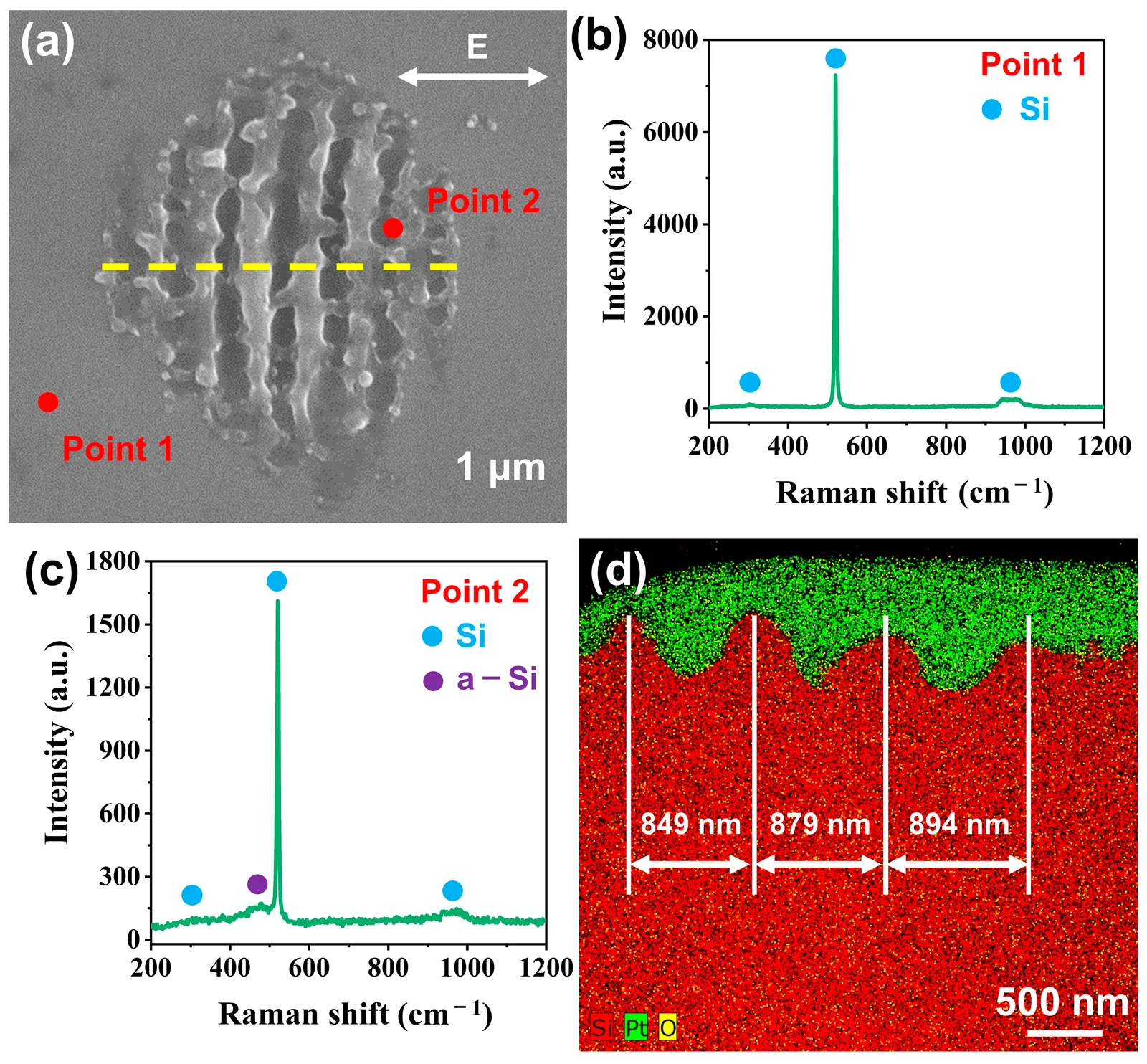
(**a**) The SEM morphology of the LIPSS; Raman spectrum results of (**b**) unirradiated Si, and (**c**) LIPSS; (**d**) EDS map of the FIB lamella extracted from the yellow dashed line in (**a**).

Figure 4a displays the SEM image of the LIPSS induced by fs laser with a laser fluence of 0.25 J/cm^2^, exhibiting a state perpendicular to the horizontal polarization direction of the laser beam. Surface melting usually occurs in fs laser-induced Si structures in the air [2]. Figure 4b,c provide the Raman spectroscopy analysis of the unirradiated Si (Point 1) and the LIPSS (Point 2). As shown in Figure 4c, three intrinsic Raman peaks located at around 300 cm^−1^, 520.5 cm^−1^, and 960 cm^−1^ are observed, which belong to two transverse acoustic (2TA) modes, transverse optical phonon (TO), and two transverse optical (2TO) modes, respectively [41]. As shown in Figure 4c, in addition to the intrinsic Si peak, a broad a-Si peak appears at 480 cm^−1^ in LIPSS, which is a good indicator of laser irradiation-induced melting of the Si surface. Furthermore, the peak intensity of the TO mode measured from the LIPSS is significantly lower than that measured from the unirradiated Si, implying a high absorptivity or intense scattered laser [42].

The EDS analysis of the cross-sectional morphology of the FIB lamella is presented in Figure 4d. Both the LIPSS and the deposited Pt layer are observed. The overall profile of the LIPSS in Figure 4d presents that the average period of the typical subwavelength structure within LIPSS is around 874 nm, which is close to the calculated period shown in Figure 3d. The apparent asymmetry of the LIPSS is attributed to the slight deviation of the incident laser beam from the normal direction of the sample. The superposition of Si, O, and Pt elements shows that the crystalline Si is below the LIPSS, and the Pt layer deposited on the top of the structure leads to a higher content of the O element. There is no significant O accumulation between the Pt layer and the crystalline Si layer, which means no evident redox reaction during the formation of the LIPSS. The laser-induced amorphization and atomic structure of the LIPSS obtained above are further confirmed and visualized in the cross-section of the irradiated Si structure described below.

### 4.3. Phase Transition Mechanism

**Figure 5:**
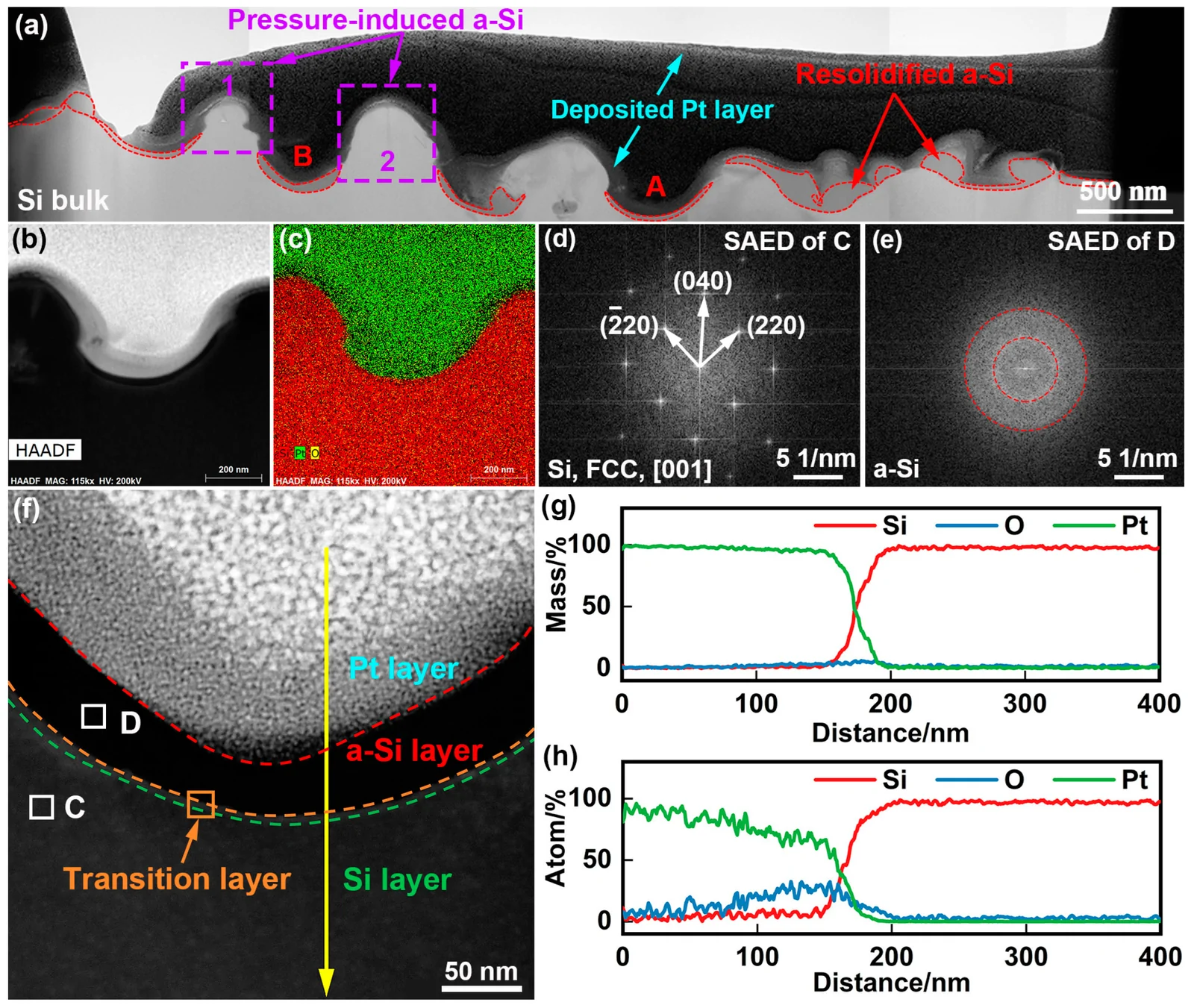
TEM analysis of the LIPSS. (**a**) TEM bright-field image (red dashed lines indicate the a-Si region), numbers 1 and 2 represent regions of interest in Figure 7; (**b**) HAADF image of the region A in (**a**); (**c**) superposition of Si, O, and Pt elements; (**g**) HAADF image of region B in (**a**); (**d**,**e**) SAED patterns of regions C and D in (**f**); (**g**,**h**) EDS line scan analysis of the yellow line in (**f**).

Figure 5a illustrates the overall cross-section morphology of the LIPSS, which is clearly visible under the dark deposited Pt layer. The central region of the LIPSS has a larger structure size and a low position, categorizing it as a highly modified region. However, the laser energy received by the edge regions is relatively low. This region is categorized as a lower modification region with a high position and smaller structural size. According to the LIPSS morphology in Figure 5a, it can be found that the surface damage distribution is highly complex. Therefore, TEM observation was used to further reveal the damage evolution mechanism induced by fs laser processing.

**Figure 6:**
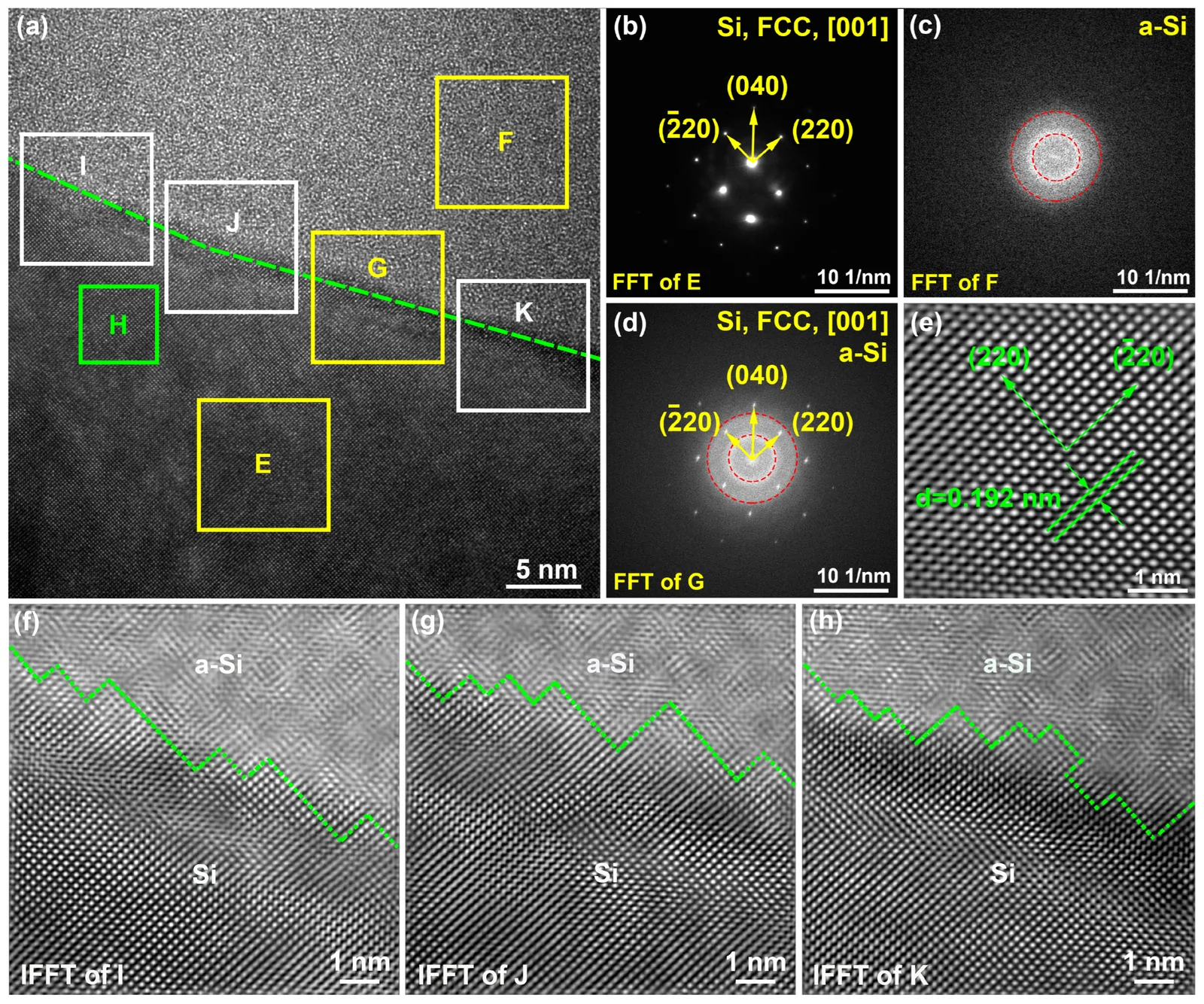
HRTEM analysis of the transition layer of Figure 5f. (**a**) HRTEM image of the Si and a-Si interface; FFT images of regions (**b**) E, (**c**) F, and (**d**) G; (**e**) IFFT image of region H; IFFT images of regions (**f**) I, (**g**) J, and (**h**) K.

Figure 5b indicates the high angle annular darkfield (HAADF) image of region A in Figure 5a. It can be observed that the O content in the highly modified region is extremely low from the EDS maps results in Figure 5c. Moreover, the Si region beneath the LIPSS is not affected by the laser. Figure 5f demonstrates the HAADF image of region B in Figure 5a. The EDS-line scan results in Figure 5g and h corresponding to the yellow line in Figure 5f prove the low O content in the LIPSS. Figure 5d exhibits the SAED pattern of region C in Figure 5f, and the calibration result suggests that it is the [1] ZA of Si, which consists of (220), (220), and (040) lattice planes. The diffraction spots are entirely attributable to the Si crystal, i.e., there is no sign of oxidation forming the SiO_x_, indicating that no oxidation of molten Si occurred during the resolidification process. Furthermore, the amorphous ring demonstrated in Figure 5e corresponds to the SAED pattern of the region D in Figure 5f, which indicates an a-Si layer with a thickness of 50 ± 10 nm caused by melting and ultra-fast solidification. Due to the low-energy deposition of the first Pt protective layer, the amorphization induced by Ga^+^ ion irradiation during the FIB sample preparation can be ignored. Moreover, a transition layer with a few nanometers thickness is observed between Si and a-Si layers (see Figure 6). This phenomenon states that both the two phases could coexist. Therefore, the LIPSS in Figure 5f can be divided into the a-Si layer, transition layer, and unaffected Si layer from the peak to the bottom. The a-Si layer in Figure 5a is marked and surrounded by red dashed lines, indicating that the amorphization of Si occurs during LIPSS formation.

Fs laser-induced phase transitions involving the crystal structure of Si are evidently presented at the atomic scale using HRTEM. Figure 6a displays the HRTEM image of the transition region (orange square) in Figure 5f. Figure 6b–d present the detailed FFT images of the regions E, F, and G, respectively, confirming the existence of Si and a-Si in the transition layer. Moreover, the detailed IFFT image in Figure 6e illustrates that region H maintains a perfect face-centered cubic (FCC) structure, that is to say, the unaffected Si layer remains its initial structure and was not affected by the fs laser. Figure 6f–h indicate the IFFT images of the regions I-K in Figure 6a. It is seen that the a-Si layer is located above the boundary line (green dash line), exhibiting typical short-range order and long-range disordered amorphous characteristics. In contrast, the Si region below the boundary line was not affected by the fs laser, which was consistent with Figure 6e. In particular, defects such as dislocations and stacking faults are not observed at the Si/a-Si interface, indicating that the relatively smooth interface ensures the tight connection between the crystal Si and a-Si layers during the LIPSS formation, which is consistent with the previous study [43].

**Figure 7:**
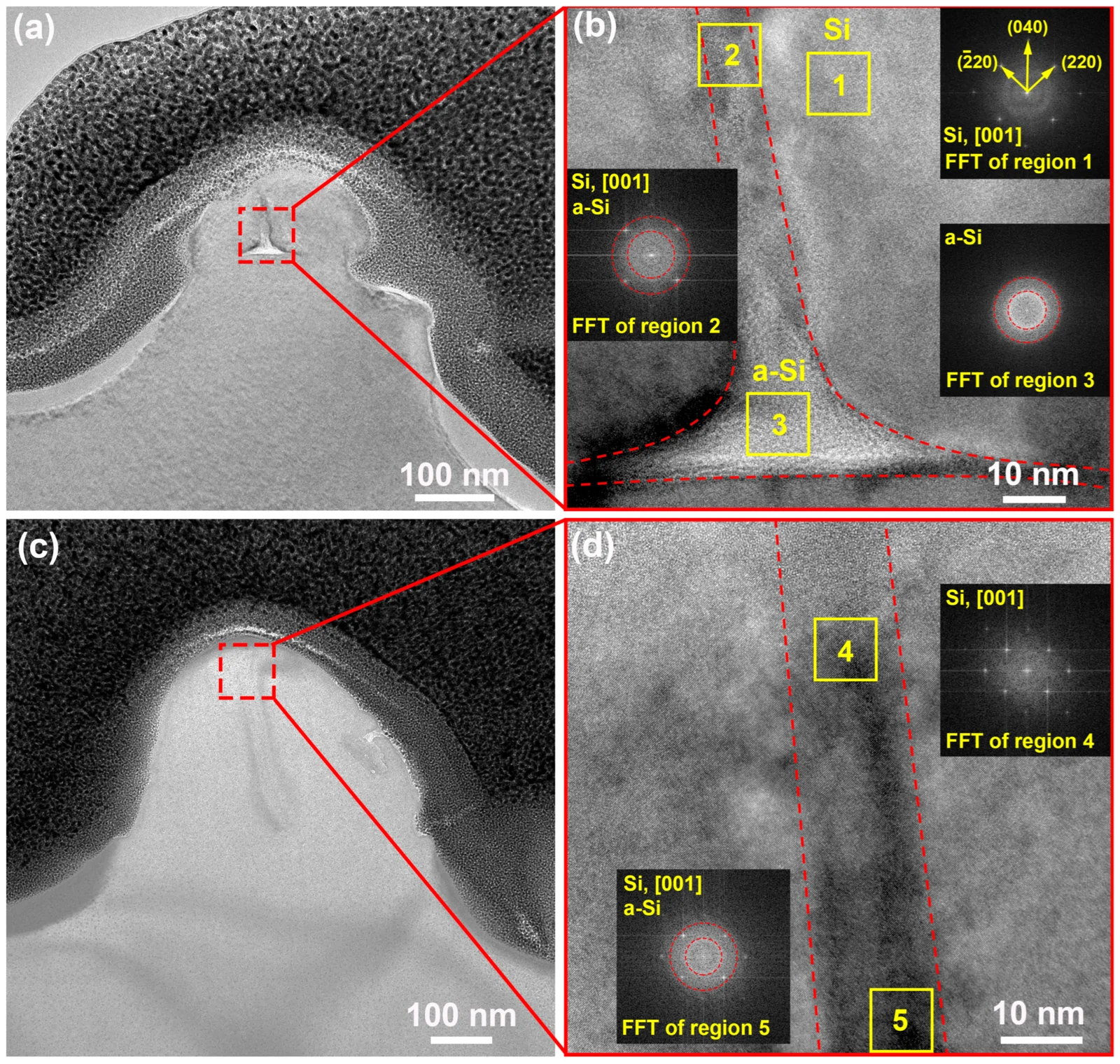
(**a**) Bright-field image of the purple dashed square 1 in Figure 5a; (**b**) HRTEM image of the red dashed square in (**a**); (**c**) bright-field image of the purple dashed square 2 in Figure 5a; (**d**) HRTEM image of the red dashed square in (**c**).

The formation of surface structure induced by fs laser processing is commonly accompanied by a series of phase transitions on the subsurface of the materials [44,45]. To further analyze the phase transition of the subsurface layer of the LIPSS, HRTEM analysis was performed on the purple square 1 in Figure 5a. The amorphous transition phenomenon in the subsurface layer near the left peak of the LIPSS was also observed. Figure 7a shows the bright field image of the purple dashed square 1 in Figure 5a, indicating that an inverted “T”-shaped region was generated at about 100 nm beneath the peak. The HRTEM image in Figure 7b, referring to the red dashed square in Figure 7a, represents the a-Si region. Moreover, the detailed FFT images corresponding to regions 1, 2, and 3 indicate that the amorphous region exhibits an increasing degree of atomic disorder from the peak to bottom regions, reflecting the gradual increase in the degree of amorphization. The bright-field image in Figure 7c exhibits a similar amorphous region corresponding to the purple dashed square 2 in Figure 5a. According to the detailed FFT images of regions 4 and 5 in Figure 7d, the structure of this region is similar to the a-Si region shown in Figure 7a, i.e., the amorphization becomes evidently from the upper region to the lower region.

The previous report has demonstrated the ability of fs laser to induce the amorphous top layer on Si with thicknesses on the order of several 10 nm [46]. Moreover, the coherent superposition of incident fs laser can promote periodic localized melting and amorphization processes of Si, as well as enhance energy deposition [40]. Meanwhile, a moving fs laser can induce LIPSS based on nonthermal melting and rapid solidification amorphization of Si depending on the free electron density. Unlike existing reports, single-pulse processing was used in this study, and the amorphization of Si was achieved by an extreme nonequilibrium process caused by fs laser irradiation. The laser photons transferred energy to electrons within 450 fs, resulting in a dramatic increase in the free carrier density. Subsequently, the electrons and the lattice gradually increased the lattice temperature within 6 ps through the coupling effect. During this period, the periodic oscillating energy field ablation formed by the interference of SPP and incident fs laser leads to the formation of the LIPSS. Finally, the a-Si layer is formed on the LIPSS surface due to the ultrafast solidification effect [47,48]. Nevertheless, the formation mechanism of the a-Si region located in the subsurface of the LIPSS is different from that of the a-Si layer on the LIPSS surface. Fs laser irradiation could trigger the required pressure to drive the phase transition of Si [49,50]. Firstly, the Si lattice is heated up in an extremely short time, and a thermoelastic pressure generates during the fs laser irradiation process. Then, the Si layer is completely gasified and rapidly expanded when the absorbed energy reaches the ablation threshold. The plasma expansion drives the recoil shock wave into the Si substrate. Finally, the residual stress is generated within the remelted Si after fs laser irradiation [51,52]. In addition, the volume expansion of the thin molten layer produces tensile stress at the bottom of the groove and compressive stress at the sides [53]. According to Figure 7, the a-Si region is only formed at the center of the protruding peak, indicating that the pressure generated on the surface is not uniform. This region is expected to be in a compressed state. Moreover, Figure 5a shows that the tips of the peaks on both sides of the LIPSS are also covered with an a-Si layer, indicating an intermediate state of a-Si during the formation of the LIPSS. Therefore, the pressure-induced phase transition of the a-Si region formed in the subsurface of the LIPSS is driven by the solidification-induced stress [54,55].

**Figure 8:**
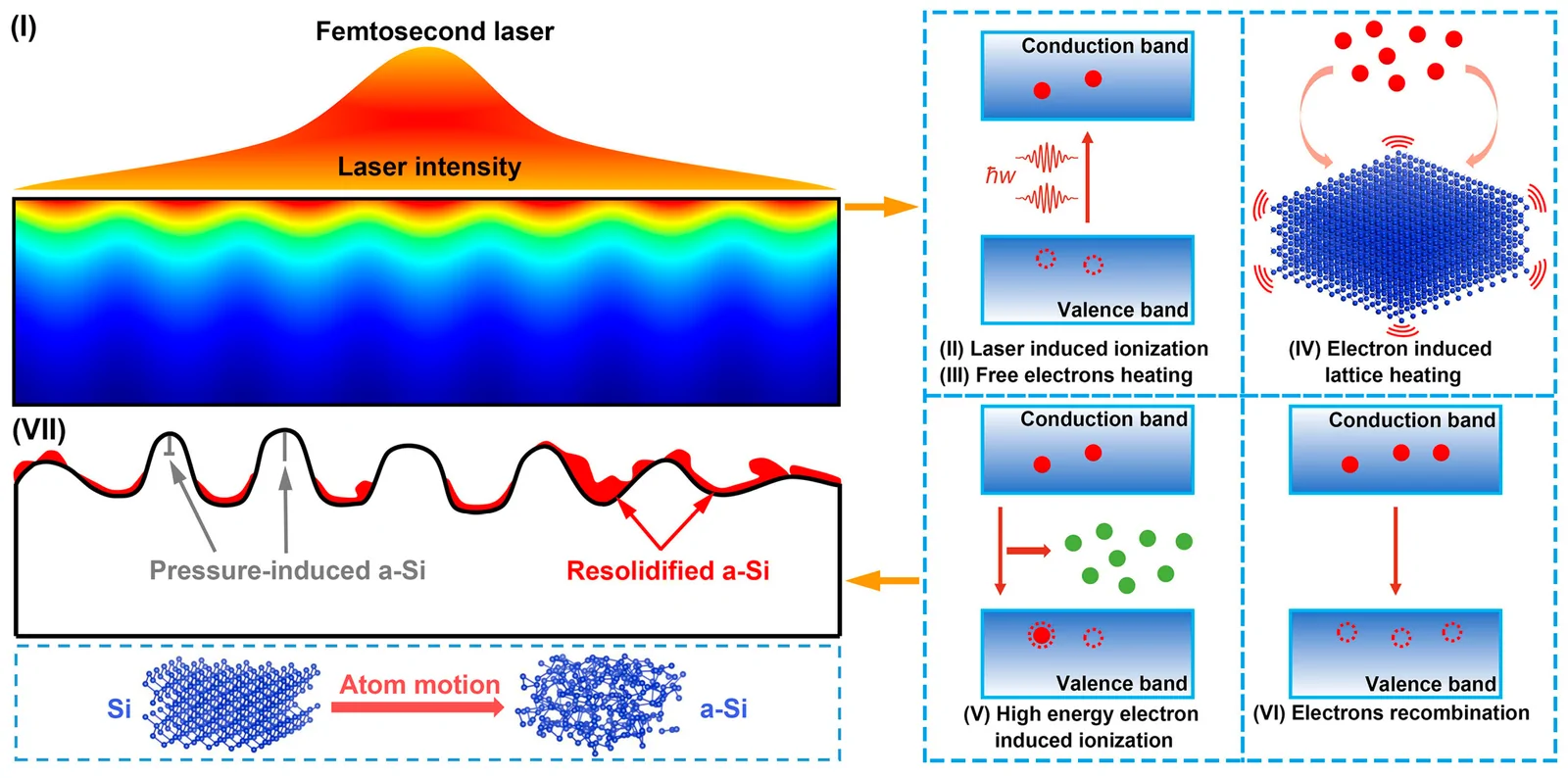
Scheme of electron and lattice dynamics interactions during LIPSS formation.

Based on the experimental and simulated results, a model for electron and lattice dynamics interactions during LIPSS formation is proposed, as displayed in Figure 8. The interaction process between fs laser and Si can be described as follows:

[I] The periodic distribution of incident fs laser energy in space;

[II] Laser-induced ionization: nonlinear ionization of electrons was excited by fs laser, involving single-photon and two-photon absorption;

[III] Free electron heating: inverse bremsstrahlung-induced in-band heating transfers energy to free electrons;

[IV] Electron-lattice coupling effect induced lattice heating: energy transfer from free electrons to the lattice to cause lattice instability and increase lattice temperature to achieve thermal melting [56];

[V] Electron-induced ionization: free electrons with high kinetic energy in the CB collide with bound electrons, resulting in ionizing multiple free electrons;

[VI] Recombination of holes and electrons in the VB;

[VII] LIPSS formation and amorphization: the periodic oscillation energy field ablation formed by the interference of SPP and incident laser leads to the LIPSS formation. The a-Si layer is formed by ultrafast nonequilibrium solidification, and the solidification-induced stress drives the generation of subsurface amorphization.

## 5. Conclusions

In summary, the electron (lattice) dynamics and micro-nano structure of Si under fs laser irradiation have been deeply studied from the aspects of numerical simulations and experiments in this work. The main conclusions are as follows.

(1) The modulation of laser energy results in the periodic distribution of electron (lattice) and micro-nano structure. The carrier density affects the optical properties of Si during laser irradiation.

(2) Three distinct layers were found in the LIPSS: an amorphous silicon layer, a transition layer, and an unaffected Si layer. The a-Si layer is located on the surface, and the atomic structure is characterized by short-range order and long-range disorder. The unaffected Si layer is not affected by the laser irradiation and maintains the initial perfect FCC structure. The transition layer is a mixed region of the a-Si and unaffected Si layers.

(3) The a-Si layer on the LIPSS surface is generated by ultrafast solidification, while the a-Si region on the LIPSS subsurface is caused by the stress induced by the solidification of molten Si.

This study provides a new perspective for investigating fs laser processing of Si and provides guidance for the preparation of semiconductor surface micro-nano structures and sub-surface structure analysis. Furthermore, beyond the case of a single laser pulse processing, it is valuable to extend to a detailed microscopic investigation of multi-point induced effects.

## Figures and Tables

**Table 1:** The parameters used in the simulation [32].

Symbol	Nomenclature	Values	Unite
*C*_A_	Auger recombination coefficient	3.8 × 10^−43^	m^6^/s
*C*_e_	Electron specific heat capacity	3*k*_B_*N*_e_	J/(m^3^·K)
*C*_l_	Lattice specific heat capacity	$1.978\times 10^{6}+3.54\times 10^{2}T_{l}-3.68\times 10^{6}T_{l}^{-2}$	W/(m·K)
*c*	Light speed	3 × 10^8^	m/s
*e*	Electric charge	1.6 × 10^−19^	C
*E*_0_	Bandgap energy	$1.16-7.02\times 10^{-4}T_{l}^{-2}/\left(T_{l}+1108\right)-1.5\times 10^{-8}{N_{e}}^{1/3}$	eV
*J*_e_	Electron generation	$\frac{\psi_{1}I}{\hbar \omega }+\frac{\psi_{2}I^{2}}{2\hbar \omega }+\delta N_{e}$	(m^3^·s)^−1^
*k*_e_	Electronic thermal conductivity	$2k_{B}^{2}\mu_{e}N_{e}T_{e}/e$	W/(m·K)
*k*_l_	Electronic thermal conductivity	$1.585\times 10^{5}{T_{l}}^{-1.23}$	J/(m^3^·K)
*m* ^*^	Effective optical mass	1.64 × 10^−31^	kg
*N*_t_	Threshold density for electron-photon coupling	6.02 × 10^26^	m^−3^
*N*_0_	Total valance band density	2 × 10^29^	m^−3^
*n*_0_	Atomic density of Si	5 × 10^28^	m^−3^
*P*_e_	Auger recombination	$N_{e}/\left(\tau_{m}+{\left(C_{A}N_{e}^{2}\right)}^{-1}\right)$	m^−3^
*t*_p_	Pulse duration	300	fs
*μ*_e_	Carrier mobility	$e/\left(m^{*}\nu \right)$	m^2^·V^−1^·s^−1^
ν	Carrier collision frequency	7.87 × 10^14^	Hz
λ	Wavelength	1030	nm
ω	Laser angular frequency	2πc/λ	s^−1^
$\omega_{p}$	Plasma frequency	$\sqrt{N_{e}e^{2}/m^{*}\varepsilon_{0}}$	s^−1^
ϒ	Energy coupling rate between electron and lattice	$C_{e}/\tau_{\gamma }$	W/(m^3^·K)
$\tau_{\gamma }$	Electron-photon coupling time	$\tau_{\gamma }^{0}\left[1+{\left(N_{e}/N_{t}\right)}^{2}\right]$	s
$\tau_{\gamma }^{0}$	Minimum electron-photon coupling time	2.4 × 10^−13^	s
$\tau_{m}$	Minimum Auger recombination time	6 × 10^−12^	s
ξ	Modulation amplitude	0.15	-
$\psi_{1}$	One-photon absorption	1.021 × 10^5^	m^−1^
$\psi_{2}$	Two-photon absorption	1 × 10^−10^	m/W
δ	Impact ionization coefficient	$3.6\times 10^{10}\exp\left(-\frac{3}{2}\frac{E_{0}}{k_{B}T_{e}}\right)$	s^−1^

## Data Availability

Data available on request due to restrictions eg privacy or ethical.
