# Correction: Liu et al. Research on Monocrystalline Silicon Micro-Nano Structures Irradiated by Femtosecond Laser. *Materials* 2022, *15*, 4897

**DOI:** 10.3390/ma19183908

**Published:** 2026-09-15

**Authors:** Yanan Liu, Ye Ding, Jichang Xie, Mingjun Chen, Lijun Yang, Xun Lv, Julong Yuan

**Affiliations:** 1 School of Mechatronics Engineering, Harbin Institute of Technology, Harbin 150001, China; tjgdlyn@163.com (Y.L.); chenmj@hit.edu.cn (M.C.); 2 Key Laboratory of Micro-Systems and Micro-Structures Manufacturing, Ministry of Education, Harbin Institute of Technology, Harbin 150001, China; 3 Laboratoire Roberval, UTC, Sorbonne Universités, Université de Technologie de Compiègne, Centre de Recherche Royallieu, CS60319, CEDEX, 60203 Compiègne, France; xjctjpu@163.com; 4 Xinchang Research Institute of Zhejiang University of Technology, Xinchang 312500, China; lvxun@zjut.edu.cn (X.L.); jlyuan@zjut.edu.cn (J.Y.); 5 College of Mechanical Engineering, Zhejiang University of Technology, Hangzhou 310014, China

In the original publication [1], there were mistakes in Figures 2 and 3, as well as in their legends. The corrected Figure 2 and Figure 3, together with the corrected legends are presented below:

Correspondingly, the citations of Figure 2 and Figure 3 in Section 4.1, Paragraphs 1 and 2 have been corrected. The corrected paragraphs appear below:

Based on simulating the electron (lattice) dynamics of Si under fs laser irradiation, the evolution of the electron (lattice) temperature and optical properties can be obtained. Figure 2 illustrates the distribution of electrons, lattice temperature, and carrier density of Si at 12 ps under laser irradiation. It can be discovered that the distribution of electrons, lattice temperature, and carrier density of Si exhibit evident periodicity, which is modulated by the periodic interference energy field formed by the incident laser and the SPP. Figure 3a shows the time-dependent evolution of electron (lattice) temperature and carrier density at the x = 0, y = 0 and z = 0 positions. Firstly, the carriers absorb the laser pulse energy in an extremely short time, resulting in a rapid temperature rise to a peak value of 4.73 × 10^4^ K at 600 fs. Meanwhile, the carrier density reached a peak of 3.69 × 10^27^ m^−3^. Subsequently, the thermalized free electrons undergo rapid inelastic collisions with the surrounding lattice, which are transferred to the lattice system through the carrier–lattice coupling effect. The electron temperature and carrier density gradually decrease due to the end of laser incidence. Finally, the electron and lattice temperatures reach thermal equilibrium at 12 ps. Notably, the equilibrium temperature is approximately 6700 K, which is higher than the melting point of Si (1687 K), indicating that the laser fluence has exceeded its melting threshold.

Based on the Drude model, a large number of electrons in the VB are excited by fs laser to form free electrons in the CB. The carrier density change in Si will affect its optical properties [40]. Figure 3b displays the evolution of the real part of the dielectric constant and surface reflectivity of Si. The fs laser irradiation leads to the increasing carrier density. During this process, the reflectivity is reduced with the enhanced absorption of laser energy by Si. With the increase of carrier density, the real part of the dielectric constant gradually decreases and eventually becomes negative, i.e., the optical properties of the Si surface will evolve from a semiconductor to a metal-like material. The resonance absorption is generated when plasma frequency approaches the laser frequency, resulting in the lowest reflectivity. Subsequently, the increased reflectivity indicates a decrease in the energy coupling efficiency between the laser and the highly excited surface. Figure 3c demonstrates that the absorption coefficient increases rapidly in the high excitation state and gradually decreases when the laser pulse disappears. This result reflects that the variations of the reflectivity and absorption coefficient of Si indicate the influence of the carrier density on its optical properties. Furthermore, the wavelength range of the SPP could estimate the expected subwavelength structure on fs laser-irradiated Si. Figure 3d exhibits the period of SPP as a function of time. It is seen that the generation period varies significantly with time and the period of the SPP is in the range of 775–810 nm. Therefore, the energy transfer of fs laser irradiation on Si transmits photon energy to VB electrons through photoionization, causing changes in carrier density to affect the optical properties of Si. In particular, numerous excited free electrons form SPP on the Si surface. The energy field generated by the interference of SPP with the incident laser removes Si through an ultrafast heat effect, resulting in the LIPSS formation. Therefore, the excitation of SPP and its interference with the incident laser beam is regarded as the primary formation mechanism of the LIPSS. 

The authors state that the scientific conclusions are unaffected. This correction was approved by the Academic Editor. The original publication has also been updated.

## Figures and Tables

**Figure 2:**
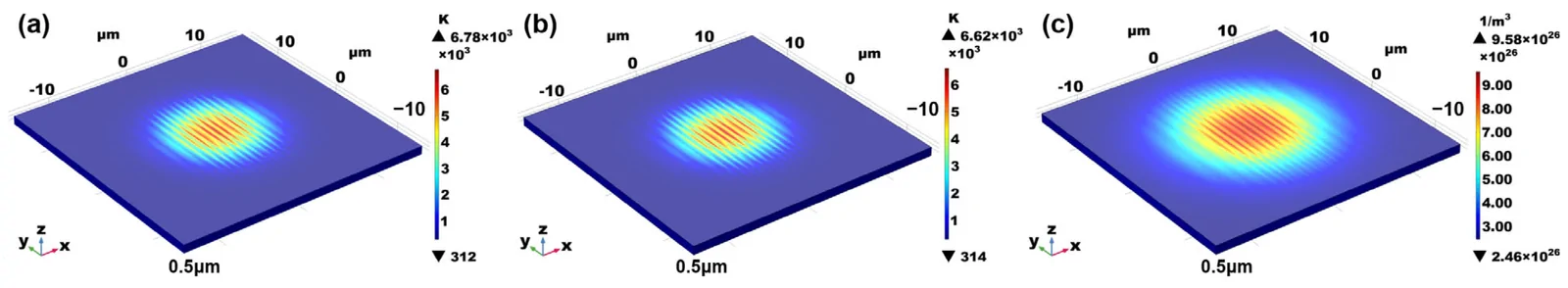
The evolution of the calculated (**a**) *T_e_*, (**b**) *T_l_*, and (**c**) *N_e_* at 12 ps.

**Figure 3:**
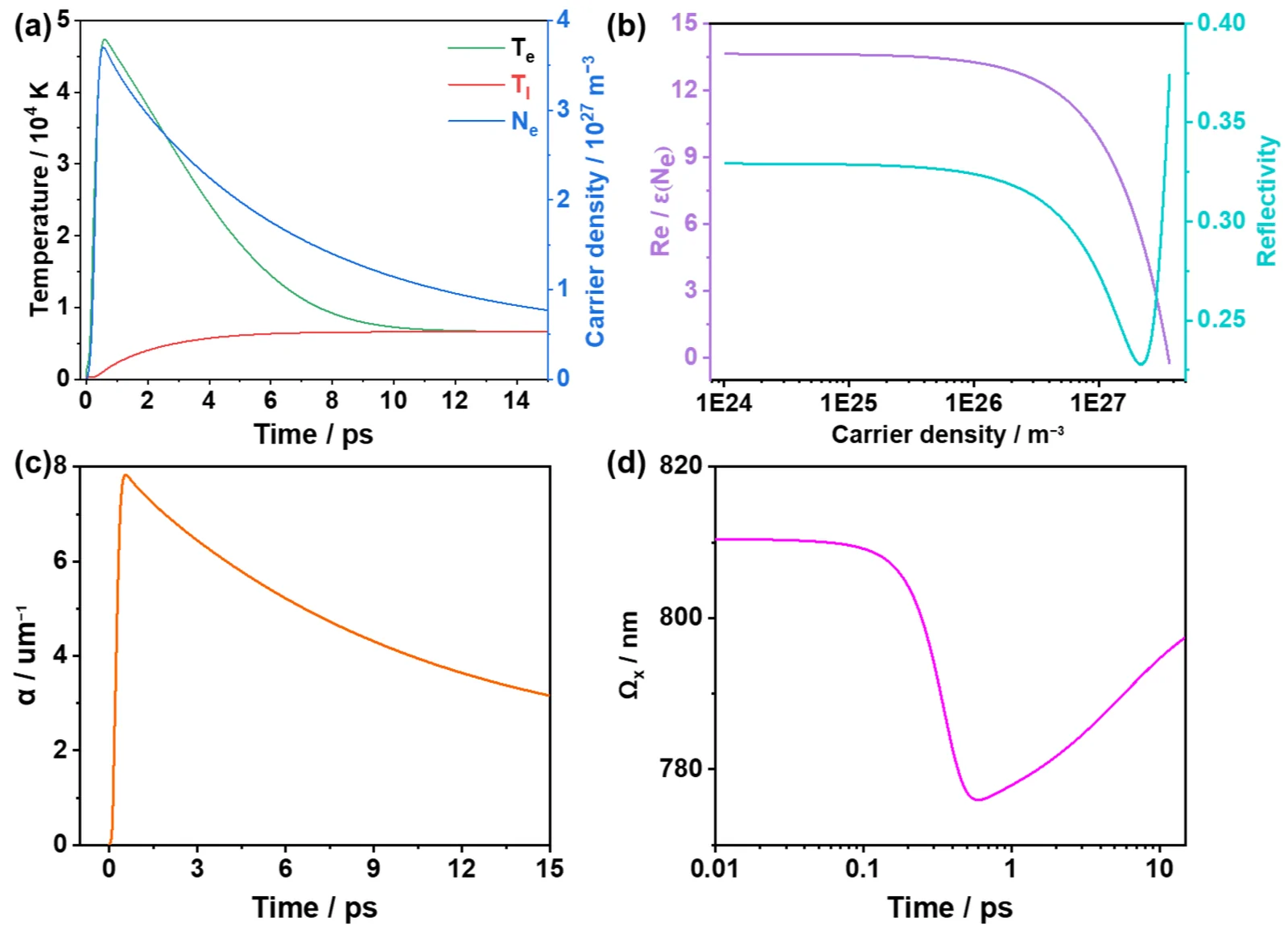
(**a**) Calculated *T_e_*, *T_l_*, and *N_e_* as a function of time; (**b**) the real part of the dielectric constant and reflectivity calculated as a function of carrier density; (**c**) time dependence of the evolution of absorption coefficient; (**d**) wavelength period of SPP as a function of time.
